# Impact role stress on turnover intentions of Pakistan’s healthcare workers: Mediating and moderating role of organizational cynicism and self-efficacy

**DOI:** 10.1371/journal.pone.0279075

**Published:** 2022-12-15

**Authors:** Tahira Nazir, Muhammad Umer, Muhammad Najam, Samina Nawab, Ahsen Maqsoom, Khuram Shafi, Yasin Munir, Iram Nawaz

**Affiliations:** 1 Department of Management Sciences, COMSATS University Islamabad, Wah Cantt, Pakistan; 2 Department of Information Technology, CareCloud, Islamabad, Pakistan; 3 Aga Khan Development Network, Islamabad, Pakistan; 4 Department of Civil Engineering, COMSATS University Islamabad, Wah Cantt, Pakistan; 5 Department of Business Administration, GC Women University, Sialkot, Pakistan; 6 Office of Career Services & Corporate Linkages, University of Lahore, Lahore, Pakistan; St John’s University, UNITED STATES

## Abstract

Nurses make up most of the global healthcare system, thus justifying their significance in the respective industry. The healthcare profession is amongst the very few careers that are attributed to a very high level of stress and imbalanced work-life equilibrium. Over past decades, the said nature of work has been observed to coerce countless nurses to leave their respective organizations. Considering this, the current study primarily evaluates the impact of role stressors on the turnover intentions of nurses employed in the healthcare industry of Pakistan. Secondly, the study examines the mediatory role of organizational cynicism between the role stressors and turnover intentions to explain the escalating trend of nurses intending to leave the industry. Lastly, it assesses the moderating role of self-efficacy between the organizational cynicism and the intended turnover of a nurse, to gauge the amount of variation self-efficacy can cause to mitigate the negative attitudes of employed individuals. The current study was conducted in the twin metropolitans of Pakistan i.e., Rawalpindi and Islamabad. A total of 394 responses were statistically evaluated using SmartPLS 3.0. The results of the current study indicated a significant impact of role stressors on the turnover intentions of nurses. Also, organizational cynicism was observed as a significant mediator between role stressors and turnover intentions. Further, self-efficacy as well was observed as a significant moderator between organizational cynicism and the intent of healthcare workers to leave the organization. The present study addressed the conceptual research gap by exploring the direct cumulative effect of role stress (role ambiguity, role conflict, role stressors, work-family conflict) in developing the intents of professionals to quit their employing organizations, the mediatory role of organizational cynicism, and the moderation effect of self-efficacy between the undertaken variables. While in terms of abridging the contextual research gap, the current study evaluated the proposed research model within the healthcare sector of Pakistan. The findings of the current study commended the management personnel of the healthcare industry to provide the nurses with healthy professional environments to work in, as well as catering to their professional and personal expectations to a better extent. Hence, increasing the ownership of an individual depicted towards his/her employing organization.

## Introduction

The availability of healthcare services is one of the most critical needs of any given society. Considering this, the effective and efficient delivery of healthcare services can only be assured with the availability of professionally qualified nurses [1]. At the same time, it also must be taken into account that for any given healthcare system around the world employed individuals are required to perform under strenuous situations. Since the quality of services and care delivered by the nurses is critical and has the well-being of the patients dependent upon it [2]. Considering which, over the past decades it has been observed that nurses often get to experience stress beyond their professional capacity. As result, potential candidates looking forward to joining the healthcare sector are reluctant to join the said industry and an increasing number of already employed individuals are leaving the profession [3]. This trend can very much be observed in a recent report by the WHO (World Health Organization) that indicated a global deficit of 7.2 million nurses, which is expected to surge up to 15 million by the year 2030 [4]. In a similar context, the research conducted in Pakistan’s healthcare industry reported a shortage of 2.2 million nurses within the country [5,6]. Both of the reports indicated the fact that nursing as a profession gets critically affected by the human resource shortage and it being a significant part of the health care sector further compromises the quality of services delivered to the patients. Drennan and Ross [5] revealed that in developed countries, on average, one nurse is available to 50–60 people, whilst in Pakistan the figure is as high as 1500 for every nurse. Alarmingly these numbers are only expected to increase in years to come. Though most of the studies conducted on adversities faced by healthcare sector are focused largely on developed regions, despite situation seems less different in a developing country like Pakistan. As result, the present nursing shortage and high rate of turnover intentions among nurses have become the dilemma for most countries including Pakistan.

The dominance of nursing staff in terms of count and role in their profession makes them indispensable to the healthcare sector. Their broad and multifaceted work pressure is characterized by a variety of role stressors, yet the profession has barely been recognized as stressful and demanding on a broader scale [7]. Nurses experience a variety of work stressors, including but not limited to shiftwork, burdening workload, long working hours, and conflicting job demands The earlier literature only has identified a few factors of role stress like work-family conflict and role ambiguity, which can potentially develop various negative attitudes within an individual including cynicism that may eventually instigate intents to turnover [8–10]. Similarly, several past studies have reported work-family conflict and job stress to have a directly proportionate association with turnover intention of an employed individual [11–13]. Considering the development of adverse attitudes because of afore mentioned role stressors, the research has indicated cynicism as the most recognizable contributor. Considering which, some of the recent studies called upon the need to have more research on role stressors and its impact on organizational cynicism and turnover intention [14,15]. Similarly, a group of researchers proposed to study different workplace stressors and their possible adverse outcomes in the organizational context [16]. Thus, justifying the need to examine the impact of role stress on organizational cynicism amongst Pakistani nurses and their overall impact on intention to quit.

Moreover, a recent study conducted by Mansour and Tremblay [17] suggested the impact of role stressors on organizational cynicism and turnover intention. Similar studies further emphasized investigating the role of self-efficacy as a moderator between role stressors and turnover intention [18,19]. As it may lead to the mitigation of adverse attitudes developed in an individual caused by role stressors.

Various researchers have a consensus on assessing the role of self-efficacy in association with work stress while considering its role as a moderator [20]. Evaluating self-efficacy specifically in the context of the nursing profession has so far revealed that the individuals who perceive themselves as more efficacious in their professional routine experience fewer dysfunctional consequences in the working environments and face a lower level of role conflict [18]. The results of the study conducted by Laschinger, Borgogni [21] showed that nurses who had higher self-efficacy experienced lower levels of cynicism and turnover intention. Further, these individuals depicted a higher level of contentment and a soothing effect on their job.

Considering the contextual as well as conceptual research gap identified by the prior research discussed earlier, the current study aims to respond to three research questions. Firstly, it gauges the effect of role stressing events that trigger antagonistic outcomes such as an intention to quit the employing organization, while considering the cumulative effect of all of its dimensions i.e. (role ambiguity, role conflict, role stressors, and work-family conflict). Further, this study evaluates the mediating effect of organizational cynicism on the relationship between the role stressor and turnover intentions. Lastly, it assesses the moderating role of self-efficacy between organizational cynicism and the turnover intention developed in the nurses of Pakistan. In specific to addressing the contextual research gap the present study evaluates the hypothesized model in the healthcare sector of Pakistan, which priorly has remained a less prioritized contextual domain to be explored.

The following sections of the study are composed of a detailed literature review of the undertaken variables and their proposed relationships. Further, the methodology adapted to collect the data from the targeted population is discussed, which is then followed by the statistical analysis of the collected data. Lastly, the managerial deductions based upon the attained information are quoted and concluded in the discussion and conclusion section.

## Literature review

### Role stressors

The theory of role stress suggests that an individual may experience stress after performing in a particular role for a number or reasons. It either can be because of the mismatch between the role expectations and one own potential, ambiguity regarding the assigned role, the conflict between the multiple roles one is performing in parallel to one another, or compromising overlap between the role demands. All of these aspects may result in a person becoming incapacitated in terms of fulfilling assigned job roles and developing physiological/psychological adversities [22].

Potentially, harmful reactions and aversive strains associated with individuals are often led by environmental factors called role stressors. Role stressors are the strain factors that hinder an individual to perform effectively and efficiently not only professionally but also in a personal capacity. Role stressors experienced in organizational settings are divided into four dimensions i.e., role conflict, role ambiguity, work-family conflict, and role overload [23–26].

Beginning with the first dimension of role stressors, role conflict in an organizational setting suggests that professional roles are interlinked, and the uncertainty associated with their diversification may result in a dispute. The said interdependence of roles not only creates conflicts among employed individuals but also an inability to fulfill the desired expectations related to those roles. Role conflict mostly arises upon experiencing incompatible or inconsistent pressure of work associated with unrealistic job demands and deadlines [25,27]. Secondly, role ambiguity occurs in the absence of a clear agenda and the knowledge required to perform the duty. In an organizational environment, role ambiguity reflects the degree of employees’ uncertainty regarding the appropriate actions required to accomplish job functions. As a result, when facing role ambiguity, workers experience stress as they constantly have a fuzzy picture of their professional responsibilities, their peers’ and supervisor’s outlook of them. Role ambiguity may also arise due to the magnitude of intricacy associated with the job [25,28]. Thirdly, the aspect of role overload refers to the exertion of too much effort in the specified time. Similarly, few researchers consider role overload as a form of stress when individuals feel incompatible to meet deadlines and do have not adequate time and energy to accomplish undertaken tasks [26,29]. As Katz and Kahn [30] affirmed that role overload appears when excessive workload has burdened the individuals within the constraint of time and demands exceed the potential. Moreover, role overload refers to the perception of individuals having multiple expectations from the authority and they feel incapacitated to complete the expectations comfortably. Considering which, prior research has indicated that employees leave their organizations due to intolerable stress in the form of role overload [31,32]. Some studies identified that work overload signaled most of the psychological and physiological stress which leads the employees towards physical illnesses as severe as chronic heart diseases [33,34]. Lastly, the work-family conflict is defined as a form of inter-role conflict in which the role pressure associated with the work and family gets imbalanced. That is participation in the work role is compromised by the act of participation in the family role and vice versa. The two primary domains in the daily life of all adults are home and work. Therefore, in an ideal situation, no conflicts are supposed to overrun the two domains affecting each other, but o the situation is quite opposite. Work-family conflict evolves when either domain affects the other [35,36]. The family-work conflict occurs when the family issues affect the work-life of an individual. Conversely, work-family conflict occurs with the negative effect of work adversely influence family life [37,38].

Referred to the classification of role stressors, role ambiguity, and role conflict are among chronic job stressors which are researched most frequently, because of their critical nature and common incidence in organizational environments. Their chronic attribution is because of their commonly observed infliction upon individuals at any workplace [39]. Further, there are short-term stressors that are usually referred to as acute or event stressors. These stressors can be as ordinary as patients being encountered by a nurse for the first time, as annoying as computer shutdowns experienced by a research assistant, or as dramatic as an armed suspect encountered by police [40]. Lastly, occupational or work-related stressors include role overload, role ambiguity, and role conflict. These are the three out of four dimensions of role stress that are specifically related to the healthcare staff [41–43].

Considering which, Colligan and Higgins [44] reported a directly proportionate relationship between the consequences of stressors with psychological disorders, intent to leave, declined organizational commitment, and job satisfaction. Moreover, role stressors adversely affect the work-life balance and overall health of employees. Which further adds up to the overall cost of the healthcare services delivered [45]. Regarding monitoring the consequences and causes of role stressors, management of healthcare organizations are more concerned now than ever. Over the years research has persistently been indicating inclined stress levels amongst healthcare employees. The referred phenomenon is of most critical consideration in the healthcare industry than in any other profession since it involves consequential stakes as high as a patient’s life dependency [46].

The stress associated with the nurses can be observed on all the functional levels of employment, either by a fresh graduate entering the organization or an experienced professional in the industry looking forward to retiring next year. Altier and Krsek [47] reported the experience of new medical graduates indicating the induced values and clinical work roles being different from those learned in school. This further led to cause role overload and role ambiguity among the newly registered nurses upon their entry into a hospital environment. Similarly, Chang, Hancock [48] and Admi and Eilon-Moshe [49] indicated role overload as the main source of stress for nurses. Further, Sharma, Dhar [50] elaborated that varying demands of an individual in any situation, either it be emotional, physical, or environmental could generate stress.

Role stressors generally stem from various aspects like role conflict, role ambiguity, work-family conflict, work relationship, task condition, structure, and climate of the organization, etc. Conclusively, the conceptualization of role stressors renders a negative impact on an employee that can potentially result in various adverse forms such as the development of turnover intention [51–53].

### Relationship of role stressors and turnover intention

Various studies have indicated that role stressors can lead to the development of employees’ turnover intention. For instance, Janssen, De Jonge [54] noted that a stressful working environment not only results in lower job involvement but also leads to increased intention to leave the organization. Similarly, Hom and Griffeth [55] noted job stress as an antecedent of employee withdrawal intentions. Further, Arshadi and Damiri [56] concluded that turnover intentions are negative job-related outcomes and are caused by role-stressors. Laschinger, Borgogni [21] also indicated that stress factors in employees lead them towards turnover. The findings of Yeh and Yu [57] specified to the healthcare industry indicated that nurses have to work in a stressful environment and are influenced by workload stressors. These role stressors lead them towards turnover within the first few months of their job. These results are also aligned with the primitive theories on role stressors which suggest that conflicting or ambiguous role demands induce role strain, and this further cultivates the resignations and termination of job [58–60].

Nursing as a profession is globally predisposed to highly stressful situations, but this condition is far more critical in Pakistan. The possible reasons could be the acute shortage of nurses, as the statistics indicate that Pakistani nurse has to tackle several patients, as the ratio is for every 1500 patients, there is only one nurse to look after while in developed countries the ratio is 50–60. Akin to which, the ratio prescribed by the PNC (Pakistan Nursing Council) is 1:10 in general areas and 1:2 in specialized areas, which has been highly violated in hospital settings. As a result, more challenges in terms of nurses’ turnover are faced [5].

Literature also indicates that turnover intention is a negative outcome of job stressors. However, the relationship between job stressors and intention to leave an organization is indirectly related [61]. In a study conducted on Malaysian furniture manufacturing companies in Selangor state, 95 respondents were randomly chosen and results revealed that work stressors were positively correlated with turnover intention [62]. Considering the discussion, it can be assumed that the role stressors can potentially lead the nurses to develop turnover intention within them.

*H1*: *The role stressors will positively affect the turnover intentions of nurses*.

### Relationship of role stressors and organizational cynicism

The findings of the various studies have supported the fact that role stressors positively correlate with organizational cynicism [56,62]. Arshadi and Damiri [56] while conducting a study on 286 Iranian employees of the drilling company as well found a positive association between role stressors and cynicism. Further, Fong and Mahfar [62] indicated a significantly positive relationship between role stressors and cynicism among the employees of furniture companies. The study by Hwang, Lou [63] stated that role conflict and role ambiguity increase the level of depersonalization or cynicism associated with an employee. In another study Laschinger, Borgogni [21] discussed the stress endured by nurses and its impact on cynicism and burnout. The study further indicated that stress has a significantly inclined impact on cynicism as well as a high contribution towards the development of turnover intention. Considering the outcomes of the aforementioned studies, it can safely be assumed that role stressors can instigate cynic behavior among employed individuals.

*H2*: *The role stressors will positively affect the organizational cynicism of nurses*.

### Mediating relationship of organizational cynicism between role stressors and turnover intention

Over the years, researchers have confirmed the relationship between cynicism and role stressors [64,65]. Role ambiguity, role overload, and role conflict have been found to compromise an individual’s competence to accomplish assigned tasks. On one hand, where the accomplishment of undertaken tasks brings wellness to an individual, similarly, professional failures lead an individual to experience stress or strain. As a result, a detrimental attitude like cynicism might get developed. Further, the generalizability of such unfavorable feelings towards jobs, organizations, and peers may as well lead them towards leaving an organization [66].

It has been observed that individuals distance themselves emotionally from any effort when experiencing cynicism, which is considered a dysfunctional mode of coping with the feeling of strain [67]. Thus, minimal voluntary participation in interpersonal as well as organizational activities has been observed in highly cynical entities. When experiencing prolonged stress or severe strain, individuals develop an emotional insensitivity and cynical temperament towards their organization, work, and peers which, eventually coerce them towards developing turnover intention [66].

Shieh [68] whilst looking at cynicism in service professions reported a positive relationship between stress and cynicism. She further indicated that service professions are prone to stress and cynicism, as workers in the service industry face interpersonal interactions that are more stressful than in other professions. These stressful interactions with clients (patients) push workers to distance themselves (become cynical) from the clients they are dealing with as well as the activities that are assigned to them [69,70].

Focusing on the intention to quit several studies specific to the healthcare industry has pointed toward the presence of factors like role stress, which are responsible for an increased level of cynicism and intention to quit. As, Riahi [71] suggested that role stress amongst nurses could be tackled, to some extent, if received positive feedback from seniors (emotional acknowledgment), patients, and their families [72,73]. Possibilities like these demand efforts to be carried out in the field to mitigate role stress amongst nurses and eventually result in a decline in the number of nurses leaving the healthcare industry. As Munir, Ghafoor [74] indicated that although efforts to define role stressors have been significant, the implications of these stressors in terms of instigating organizational cynicism are yet to be explored.

The relationship between role stressors, cynicism, and turnover intentions is augmented by the above-mentioned assumptions and facts. Therefore, we can hypothesize that role stressors create negative attitudes in individuals which generate cynicism leading them to turnover intentions.

*H3*: *Organizational cynicism will positively affect the turnover intentions of nurses*.
*H4*: *Organizational cynicism will significantly mediate the relationship between role stressors and turnover intention*.

### Moderating role of self-efficacy

Various researchers have come to a consensus to explore the role of self-efficacy in relevance to its effect on work stress while considering its role as a moderator. Studying the concept of self-efficacy in the context of the nursing profession has revealed that the individuals who perceive themselves as more efficacious are probable to experience fewer dysfunctional consequences in the working environments and face a lower level of role conflict [21].

The results of Laschinger, Borgogni [21] study showed that nurses who have higher self-efficacy get to experience lower levels of cynicism and turnover intention and have a higher level of contentment while on their job.

**Fig 1:**
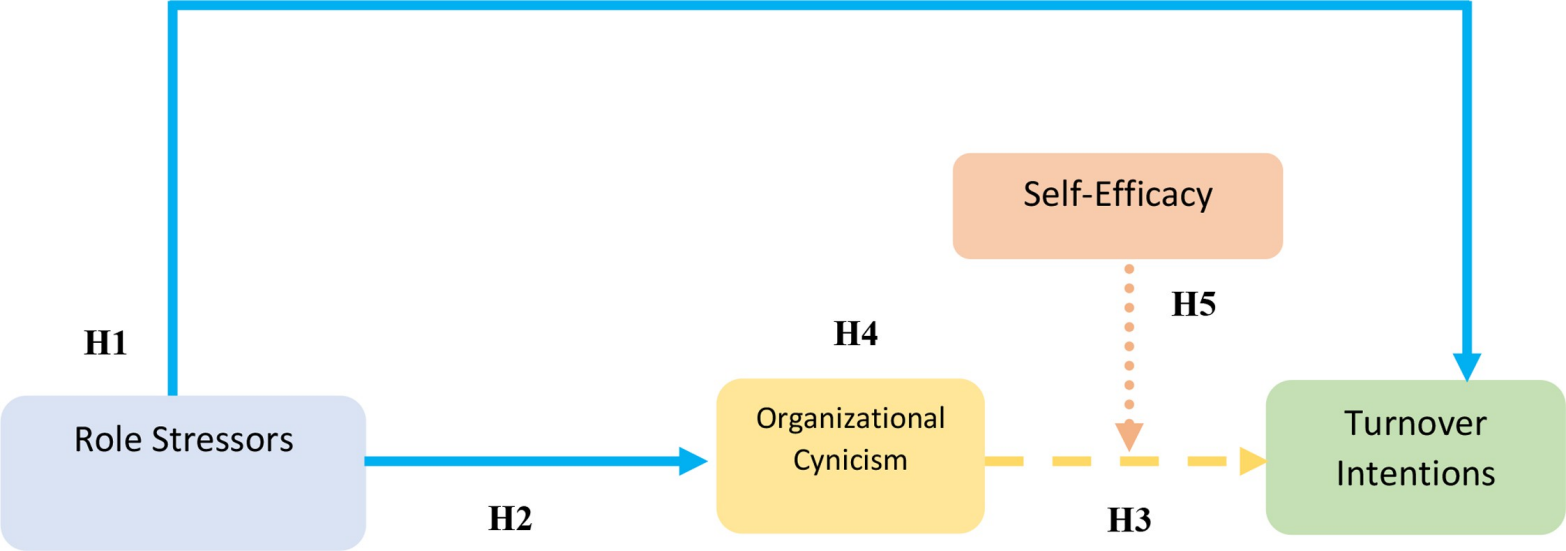
Conceptual model.

Furthermore, it has been acknowledged across various professions that the presence of self-efficacy mitigates the strain and cynicism experienced by an employed individual [75]. Consiglio, Borgogni [76] have the same opinion which suggests that self-efficacious employees are less likely to become the victim of organizational cynicism. Further, Consiglio, Borgogni [76] are of the view that self-efficacious employees shape their professional environment effectively and hence interact differently with the environment as compared to those employees who are less self-efficacious. Also, in the recently conducted study of Perrewé, Hochwarter [77] confirmed that self-efficacy could mitigate the adverse effect of role stressors especially, role conflict and role overload more effectively than any other replicating organizational behavior including organizational cynicism. Further, a longitudinal study conducted on the military personnel of Spain evaluated the impact of role stressors; work-family conflict, in particular, upon organizational cynicism while considering the buffering role of self- efficacy depicted the same results. The study further proposed prospect researchers to conduct a similar cross-sectional survey-based study on a different population [78]. Argument like these grant opportunities to examine the moderating effect of self efficacy on the relationship between organizational cynicism and turnover intentions in nursing profession, which is among the most stressful jobs in Pakistan (See Fig 1).

*H5*: *Self-efficacy will significantly moderate the relationship between organizational cynicism and turnover intention*.

## Methodology

The current study used the survey approach to collect the data from the nurses employed at various healthcare institutions in Pakistan. The development of the questionnaire involved the adaption of research items from previous studies to measure the variables of the current study i.e. (role stressors, organizational cynicism, self-efficacy & turnover intentions). The questionnaire comprised 63 items in total, with 37 items attributed to role stressors [79–82], 13 items attributed to organizational cynicism [83], 10 items attributed to self-efficacy [84], and 3 items attributed to turnover intentions [85] (See S1 Survey). Adaption of the survey items from the aforementioned studies assured the reliability and validity of the data collection instrument. The responses of the research items were recorded on a 5-Point Likert Scale, ranging from 1 depicting “strongly disagree” up to 5 depicting “strongly agree”. The current study implemented the simple random sampling technique to collect the data from the respondents. Thus, assigning every individual entity in the targeted population an equal chance to be selected. This further assures for the presented research to reflect a much truer opinion of the respondents [86,87]. A total of 550 questionnaires were disseminated among the nurses of the targeted hospitals. As a result, 394 responses got collected. Thus, justifying the response rate of 78.80%. The respondents provided their consent to use their opinion about the questioned organizational queries for scientific research purposes. The data collection process was self-supervised, which enabled the lesser chances of ambiguity in the data attained [88].

The cities targeted for the data collection procedure are the twin cities of Pakistan i.e. (Rawalpindi & Islamabad). The latter of the two is also the federal capital of Pakistan. Both cities have a diverse mix of individuals pooled from all over the country. Since these cities offer a promising future to their inhabitants as well as a better quality of life. This fact attributed the current study to a higher level of probability to attain the responses of individuals that belonged to all the provinces of the country. Thus, enhancing the generalizability of the study, in contextual terms. Moreover, the assurance of generalizability of the current study in statistical terms was justified by the fact that there are 144,600 nurses employed across the country [5], and considering the sampling criterion proposed by Zikmund, Babin [89], the required sample size for the said population is equivalent to 384. Considering this, the current study used 394 responses for testing proposed hypothesizes. Further, the collected data were statistically evaluated using IBM’s Statistical Package for Social Sciences (SPSS Version 21.0) and SmartPLS GmbH’s SMART Partial Least Squares (SMART PLS 3.0). The respective tools allowed the effective classification of the data along with its evaluation in terms of structural equation modeling.

The application of the statistical approach involved the classification of respondents in terms of their demographical attributions. Further, the demographical attributions were tested for their correlation with the rest of the behavioral variables of the measurement model i.e., IVs and DVs, to justify their contribution as a control variable in the current study. The non-significance of the correlation between the said variables discarded their consideration as control variables for the current study. The measurement model was then tested for the reliability of its adapted research items in terms of Cronbach’s alpha. The research items were further checked for their consistency in terms of factor loadings. The identified items not fulfilling the fitness criterion of factor loading were deleted from the measurement model. This approach enhanced the overall fitness of the research model. In the next step, the research items were tested for their validity (convergent & discriminant) in terms of average variance extracted, Fornell-Larcker criterion, cross-loadings, and Heterotrait-Monotrait ratio (HTMT). The multicollinearity was then checked in terms of the VIF criterion. After fulfillment of all the fitness criteria, the structural model was tested for the proposed hypothesizes (direct effects) in terms of path coefficients. Further, the overall effect-size and coefficient of determination for the structural model were evaluated in terms of *f*^*2*^ and *r*^*2*^. Lastly, the evaluation of the mediation effects and the moderation effect was tested in terms of the indirect effects and the product terms.

## Data analysis and results

The current study implemented the survey approach to collect data from respondents of various age brackets, gender, professional tenure, sector of employment, and respective employment status.

The data collected from the respondents depicted 25.6% of the individuals as males and 74.4% as females. In terms of age bracket, 51% of the individuals were aged between 20–29 years, 37.1% were aged between 30–39 years, 9.9% were aged between 40–49 years, and 2% were aged 50 years and above. The respondents showed quite a variation in terms of their professional tenure, as 22.8% had industry experience of less than 1 year, 22.8% had industry experience between 1–2 years, 27.6% had industry experience between 2–5 years, 16.2% had industry experience between 5–10 years and 10.4% of individuals had the industry above 10 years. The healthcare industry is split between the public and private sectors of the country. Considering this, 47.7% of the respondents were employed in the public sector while 52.3% were employed in the private sector. In terms of job security and employment status, 45.9% of the individual was employed permanently, while 54.1% of the individuals were employed on a contractual basis (See Table 1).

**Table 1:** Demographical classification.

Attributes		F	%
Gender	Male	101	25.6
	Female	293	74.4
Age	20–29 Years	201	51
	30–39 Years	146	37.1
	40–49 Years	39	9.9
	50 Years	8	2
Tenure	Less Than 1 Year	90	22.8
	1 ≤ X < 2 Years	90	22.8
	2 ≤ X < 5 Years	109	27.6
	5 ≤ X < 10 Years	64	16.2
	10 Years & Above	41	10.4
Sector	Public	184	47.7
	Private	206	52.3
Employment Status	Permanent	181	45.9
	Contract	213	54.1

### Application of Structural Equation Modeling (SEM)

Structural equation modeling (SEM) is a statistical technique that involves the multivariate approach to test structural relationships. Prior research has been commended to transform the interactional effects of the hypothesized model into a structural equation model [90]. The current study implemented a two-step approach to conduct SEM. The first step comprised the application of confirmatory factor analysis (CFA) to measure the uni-dimensionality of the research items associated with their respective research variables. Hair, Black [91], and Byrne [92] as well have suggested evaluating of reliability and validity of the research variables, in the first stage of SEM. The second stage of SEM involved the evaluation of the cumulative impact of the observed variables and latent variables i.e. (organizational cynicism, turnover intention, role overload, work-family conflicts, role conflicts, role ambiguity, self-efficacy) on one another; as hypothesized by the current study. This further led to the acceptance and rejection of the proposed hypothesizes.

### SEM-first stage

The first stage of structural equation modeling (SEM) involved the application of confirmatory factor analysis (CFA), to evaluate the reliability and validity attributed to the research items of each organizational variable. The evaluation of the measurement model primarily involved the detection of statistically fit research items that could be further used to test hypothesized model [92].

To begin with, the consistency of the research items in terms of collected data was analyzed upon the criterion of factor loading. This stage further involved the deletion of redundant and unfit research items. Specific to CFA, prior studies have indicated that if the values of the measurement model do not comply with the predefined fitness thresholds of the quality measures, then a model re-specification is required [93].

### Confirmatory factor analysis

To determine the reliability, validity, as well as convergence of the instruments being used in any given study, confirmatory factor analysis (CFA) is used. To consider any variable a good fit for hypothetical evaluations, prior studies have commended considering the criterion of factor loadings [94,95]. Different criteria are followed to determine the threshold of factor loadings. Kline [96] suggested that the minimum value of the factor loading associated with the item of a variable must be 0.70 to be considered a good fit. While Malle [97] and Brown and Moore [98] have suggested 0.50 and 0.30 respectively as the minimum value for the evaluation of factor loadings. The current study used the factor loading criterion suggested by Ramayah, Cheah [99], to determine the component fitness of the hypothesized model.

To begin with, the reflective-formative model was assessed for its reliability in terms of Cronbach’s Alpha. The reliability of an instrument is the determinant of its precision to measure a phenomenon repeatedly, with almost the same results. Gliem and Gliem [100] suggested the respective value of Cronbach’s Alpha for any given research instrument to be above 0.70, to declare it reliable. For the current study, the associated values of Cronbach’s Alpha for Organizational Cynicism, Turnover Intention, Role Overload, Work-Family Conflicts, Role Conflicts, Role Ambiguity, and Self-Efficacy were 0.87, 0.89, 0.91, 0.91, 0.87 and 0.83, respectively. Which justifies all the variables and their respective items to be reliable (See Table 2).

**Table 2:** Variable reliability.

Variable	Cronbach’s Alpha	Composite Reliability
**Organizational Cynicism**	0.865	0.891
**Role Ambiguity**	0.708	0.830
**Role Conflict**	0.855	0.890
**Role Stressors**	0.933	0.940
**Turnover Intentions**	0.893	0.934
**Work-Family Conflict**	0.907	0.925
**Work Overload**	0.866	0.892
**Self-Efficacy**	0.832	0.840

### Factor loadings

Factor loadings are representative of the relationship of a variable with its underlying factors. Moreover, it determines the magnitude of variability a component of a variable can cause among other associated components of a similar variable. Prior research has indicated that the value of factor loading must be 0.70 or above [101,102]. Considering which, selective items are associated with the variables of organizational cynicism (OC11, OC12), role ambiguity (RA1, RA5, RA6), role conflict (RC1), work-family conflict (WFC9), work overload (WOL 6, WOL7, WOL8, WOL 11) and self-efficacy (SE7, SE8, SE9) were found to have factor loading below 0.70 (See Table 3).

**Table 3:** Factor loadings (Stage 1).

Items	Organizational Cynicism	Role Ambiguity	Role Conflict	Turnover Intentions	Work-Family Conflict	Work Overload	Self-Efficacy
**OC1**	0.727						
**OC10**	0.722						
**OC11**	0.339						
**OC12**	0.488						
**OC13**	0.765						
**OC2**	0.743						
**OC3**	0.759						
**OC4**	0.730						
**OC5**	0.794						
**OC6**	0.772						
**OC7**	0.751						
**OC8**	0.786						
**OC9**	0.784						
**RA1**		0.323					
**RA2**		0.791					
**RA3**		0.872					
**RA4**		0.710					
**RA5**		0.157					
**RA6**		0.197					
**RC1**			0.580				
**RC2**			0.779				
**RC3**			0.776				
**RC4**			0.772				
**RC5**			0.807				
**RC6**			0.723				
**RC7**			0.760				
**RC8**			0.756				
**TI1**				0.900			
**TI2**				0.944			
**TI3**				0.878			
**WFC1**					0.765		
**WFC2**					0.737		
**WFC3**					0.828		
**WFC4**					0.796		
**WFC5**					0.749		
**WFC6**					0.837		
**WFC7**					0.735		
**WFC8**					0.738		
**WFC9**					0.623		
**WOL1**						0.768	
**WOL10**						0.755	
**WOL11**						0.395	
**WOL12**						0.779	
**WOL13**						0.727	
**WOL14**						0.710	
**WOL2**						0.724	
**WOL3**						0.725	
**WOL4**						0.709	
**WOL5**						0.798	
**WOL6**						0.558	
**WOL7**						0.279	
**WOL8**						0.565	
**WOL9**						0.738	
**SE1**							0.705
**SE2**							0.702
**SE3**							0.703
**SE4**							0.836
**SE5**							0.701
**SE6**							0.782
**SE7**							0.423
**SE8**							0.400
**SE9**							0.507
**SE10**							0.710

OC: Organizational Cynicism, RA: Role Ambiguity, RC: Role Conflict, RS: Role Stressors, TI: Turnover Intentions, WFC: Work-Family Conflict, WO: Work Overload, SE: Self-Efficacy.

The items with lower factor loadings were deleted from the initially proposed research model. Thus, increasing the fitness of the respective research variables, as well as the overall model. After the deletion of items with lower factor loadings, the model was again evaluated in the second stage of SEM.

### SEM-second stage

The second stage of SEM involved the re-assessment of the measurement model upon the measures of component and model fitness. First of which involved the evaluation based on factor loadings. The second stage of SEM depicted all the factor loading values to be well within the data fitness range. Thus, the model was considered fit for further hypothesizes evaluation (See Table 4).

**Table 4:** Factor loadings (Stage 2).

Items	Organizational Cynicism	Role Ambiguity	Role Conflict	Turnover Intentions	Work-Family Conflict	Work Overload	Self-Efficacy
**OC1**	0.738						
**OC10**	0.712						
**OC13**	0.729						
**OC2**	0.749						
**OC3**	0.764						
**OC4**	0.741						
**OC5**	0.705						
**OC6**	0.782						
**OC7**	0.755						
**OC8**	0.797						
**OC9**	0.794						
**RA2**		0.792					
**RA3**		0.799					
**RA4**		0.762					
**RC2**			0.790				
**RC3**			0.782				
**RC4**			0.776				
**RC5**			0.815				
**RC6**			0.723				
**RC7**			0.769				
**RC8**			0.757				
**TI1**				0.900			
**TI2**				0.944			
**TI3**				0.879			
**WFC1**					0.775		
**WFC2**					0.753		
**WFC3**					0.833		
**WFC4**					0.811		
**WFC5**					0.766		
**WFC6**					0.842		
**WFC7**					0.727		
**WFC8**					0.718		
**WOL1**						0.791	
**WOL10**						0.740	
**WOL12**						0.772	
**WOL13**						0.738	
**WOL14**						0.726	
**WOL2**						0.743	
**WOL3**						0.744	
**WOL4**						0.728	
**WOL5**						0.709	
**WOL9**						0.739	
**SE1**							0.718
**SE2**							0.705
**SE3**							0.754
**SE4**							0.706
**SE5**							0.780
**SE6**							0.730
**SE10**							0.712

OC: Organizational Cynicism, RA: Role Ambiguity, RC: Role Conflict, RS: Role Stressors, TI: Turnover Intentions, WFC: Work-Family Conflict, WO: Work Overload, SE: Self-Efficacy.

### Instrument validity (convergent and discriminant)

In the following stage, the research instrument and the respective dataset were tested for their validity. The validity of an instrument itself is defined as, “the extent of a given instrument to measure, what it is supposed to measure”. The validity of the measures is primarily of two types i.e., convergent validity and divergent validity. Convergent validity is defined upon the principles that, “the items of a given variable must be relatable to one another”. As it would further enable the items to represent a single variable. While on the contrary, discriminant validity is defined as “the measure to determine how unrelatable the items of one variable are to the items of the other variables” [103,104].

The convergent validity is assessed in terms of average variance extracted (AVE). The threshold for the measure of AVE is a minimum of 0.5. The constructs with values of AVE equivalent to 0.5 or higher justify the convergence of the items associated with them [105,106] (See Table 5).

**Table 5:** Average variance extracted.

Variable	AVE
**Organizational Cynicism**	0.527
**Role Ambiguity**	0.623
**Role Conflict**	0.536
**Role Stressors**	0.576
**Turnover Intentions**	0.824
**Work-Family Conflict**	0.607
**Work Overload**	0.554
**Self-Efficacy**	0.502

To determine the discriminant validity associated with the variables and their respective research items the evaluation measures of Fornell-Larcker Criterion, Cross-Loadings, and HTMT are used.

The Fornell-Larcker Criterion evaluates the variables in terms of the comparison between the square root of average variance extracted (AVE) and the correlation of the latent constructs. Considering this, it is expected of the latent constructs to explain the variance of their indicators more, than the variance of other latent constructs. For which the square root value of AVE attributed to each construct should be greater than the correlational values with other latent constructs [106,107]. Correlational values for the constructs were found fit. Thus, justifying the divergent nature of the respective constructs and their associated indicators (See Table 6).

**Table 6:** Fornell-larcker criterion.

Variable	Organizational Cynicism	Role Ambiguity	Role Conflict	Role Stressors	Turnover Intentions	Work-Family Conflict	Work Overload	Self-Efficacy
**Organizational Cynicism**	0.653							
**Role Ambiguity**	0.018	0.789						
**Role Conflict**	0.305	0.277	0.732					
**Role Stressors**	0.394	0.215	0.246	0.613				
**Turnover Intentions**	0.438	-0.044	0.318	0.422	0.908			
**Work-Family Conflict**	0.381	0.084	0.618	0.201	0.466	0.779		
**Work Overload**	0.354	0.182	0.717	0.220	0.324	0.734	0.674	
**Self-Efficacy**	0.173	0.069	0.146	0.180	0.204	0.189	0.164	0.412

OC: Organizational Cynicism, RA: Role Ambiguity, RC: Role Conflict, RS: Role Stressors, TI: Turnover Intentions, WFC: Work-Family Conflict, WO: Work Overload, SE: Self-Efficacy.

Another measure to determine the discriminant validity of the variables and the indicators associated with them is the cross-loadings. Cross-loading is evaluated in terms of the correlation existent between the variables and the factors defining it. Moreover, cross-loading is the comparative representation of the percentage variance in the variable, explained by a factor. To justify a given variable to be discriminately valid the cross-loadings associated with its indicators must value higher, in comparison to the loadings associated with other variables of the study. Moreover, the indicators must have a factor loading valued at 0.70 or above [108]. Considering the criterion of cross-loadings the variables of the current study and the associated indicators were found discriminately valid (See Table 7).

**Table 7:** Cross loadings.

Items	Organizational Cynicism	Role Ambiguity	Role Conflict	Role Stressors	Turnover Intentions	Work-Family Conflict	Work Overload	Self-Efficacy
**OC1**	0.638	-0.021	0.165	0.224	0.320	0.232	0.192	0.202
**OC10**	0.612	0.030	0.150	0.178	0.230	0.162	0.159	0.140
**OC13**	0.529	0.045	0.290	0.289	0.306	0.253	0.239	0.114
**OC2**	0.649	-0.016	0.165	0.218	0.308	0.221	0.189	0.104
**OC3**	0.664	-0.003	0.112	0.183	0.268	0.172	0.189	0.232
**OC4**	0.641	0.067	0.201	0.244	0.252	0.211	0.232	0.195
**OC5**	0.705	-0.012	0.198	0.247	0.284	0.249	0.208	0.190
**OC6**	0.682	0.020	0.214	0.255	0.267	0.239	0.225	0.240
**OC7**	0.655	-0.033	0.190	0.314	0.291	0.337	0.290	0.195
**OC8**	0.697	0.021	0.228	0.323	0.311	0.322	0.296	0.212
**OC9**	0.694	0.036	0.233	0.295	0.277	0.276	0.272	0.270
**RA2**	0.061	0.692	0.130	0.104	0.068	0.028	0.081	0.102
**RA3**	-0.008	0.899	0.270	0.226	-0.068	0.114	0.192	0.191
**RA4**	0.017	0.762	0.223	0.145	-0.061	0.025	0.126	0.126
**RC2**	0.262	0.240	0.690	0.638	0.233	0.509	0.538	0.528
**RC3**	0.193	0.125	0.682	0.570	0.208	0.422	0.476	0.468
**RC4**	0.179	0.271	0.776	0.612	0.217	0.427	0.500	0.487
**RC5**	0.251	0.275	0.815	0.660	0.268	0.441	0.576	0.462
**RC6**	0.156	0.189	0.723	0.544	0.154	0.344	0.466	0.604
**RC7**	0.278	0.179	0.769	0.692	0.276	0.519	0.610	0.604
**RC8**	0.224	0.130	0.657	0.596	0.256	0.487	0.487	0.476
**TI1**	0.391	-0.052	0.310	0.391	0.900	0.429	0.293	0.292
**TI2**	0.449	-0.047	0.300	0.386	0.944	0.423	0.295	0.296
**TI3**	0.346	-0.021	0.253	0.372	0.879	0.417	0.297	0.309
**WFC1**	0.240	-0.002	0.443	0.674	0.344	0.775	0.542	0.569
**WFC2**	0.199	0.047	0.383	0.639	0.320	0.753	0.519	0.569
**WFC3**	0.251	0.091	0.511	0.744	0.313	0.833	0.602	0.543
**WFC4**	0.294	0.062	0.417	0.689	0.378	0.811	0.553	0.543
**WFC5**	0.356	0.093	0.442	0.668	0.451	0.766	0.518	0.606
**WFC6**	0.333	0.096	0.541	0.770	0.367	0.842	0.630	0.606
**WFC7**	0.326	0.109	0.567	0.721	0.379	0.727	0.610	0.558
**WFC8**	0.370	0.017	0.530	0.696	0.353	0.718	0.588	0.558
**WOL1**	0.187	0.115	0.504	0.599	0.106	0.426	0.691	0.668
**WOL10**	0.222	0.233	0.412	0.579	0.221	0.470	0.640	0.668
**WOL12**	0.191	0.111	0.498	0.653	0.262	0.565	0.672	0.654
**WOL13**	0.266	0.067	0.523	0.712	0.225	0.626	0.738	0.654
**WOL14**	0.327	0.052	0.507	0.711	0.314	0.643	0.726	0.395
**WOL2**	0.132	0.144	0.469	0.542	0.195	0.354	0.643	0.395
**WOL3**	0.232	0.187	0.533	0.586	0.259	0.404	0.644	0.679
**WOL4**	0.187	0.154	0.412	0.535	0.081	0.393	0.628	0.679
**WOL5**	0.323	0.044	0.485	0.648	0.235	0.529	0.709	0.728
**WOL9**	0.284	0.168	0.484	0.597	0.256	0.465	0.639	
**SE1**	0.087	0.129	0.010	0.026	0.405	0.030	0.028	0.818
**SE2**	0.197	0.107	0.105	0.119	0.703	0.129	0.101	0.807
**SE3**	0.117	0.122	0.209	0.216	0.636	0.050	0.176	0.897
**SE4**	0.095	0.092	0.088	0.071	0.601	0.052	0.048	0.258
**SE5**	0.076	0.114	0.083	0.109	0.382	0.072	0.128	0.276
**SE6**	0.105	0.045	0.050	0.085	0.223	-0.001	0.088	0.384
**SE7**	0.021	0.094	0.015	-0.030	-0.200	-0.104	-0.064	0.126
**SE10**	-0.027	0.114	-0.046	0.011	0.102	-0.005	0.021	0.030

OC: Organizational Cynicism, RA: Role Ambiguity, RC: Role Conflict, RS: Role Stressors, TI: Turnover Intentions, WFC: Work-Family Conflict, WO: Work Overload, SE: Self-Efficacy.

Lastly, Heterotrait-Monotrait Ratio (HTMT) enables the determination of the discriminant validity of the variables under study as well as the indicators associated with them. HTMT is considered as a more precise criterion to justify the discriminant validity, being based upon the higher level of specificity and sensitivity ranging from 97% up to 99% as compared to the cross-loadings and Fornell-Lacker criterion which follow the precision measure of 0.00% and 20.82% respectively. Justifying the discriminant validity in terms of HTMT is based upon the comparison of evaluated correlational values to a predefined upper threshold of 0.90 [108,109]. Considering this, all the variables of the current study were found discriminately valid (See Table 8).

**Table 8:** Heterotrait-Monotrait Ratio (HTMT).

Variables	Organizational Cynicism	Role Ambiguity	Role Conflict	Role Stressors	Turnover Intentions	Work-Family Conflict	Work Overload	Self-Efficacy
**Organizational Cynicism**								
**Role Ambiguity**	0.088							
**Role Conflict**	0.344	0.334						
**Role Stressors**	0.420	0.406	0.861					
**Turnover Intentions**	0.491	0.105	0.360	0.450				
**Work-Family Conflict**	0.420	0.115	0.696	0.839	0.519			
**Work Overload**	0.396	0.234	0.829	.819	0.364	0.815		
**Self-Efficacy**	0.379	0.258	0.177	0.224	0.103	0.148	0.200	

### Assessment of reflective-formative model

The evaluation of the current study was based upon the uni-dimensional variables i.e. organizational cynicism and turnover intentions. While the study as well included the multivariate variable of role stressors which further was based upon four distinct dimensions (role ambiguity, work overload, work-family conflict, and role conflict). The inclusion of a multi-dimensional variable justified the formative nature of the variable in the measurement model. Because of this, the model was further evaluated in terms of reflective- formative criteria. This approach enabled measuring the cumulative effect of all the considered dimensions in terms of role stress.

### Collinearity evaluation (VIF)

The fitness of a formative variable is evaluated in terms of the collinearity associated with its indicators. The indicators of a given variable are expected to be correlated to some extent but not perfectly. VIF justifies the fitness of indicators of a given variable in terms of their correlational magnitude. For which the VIF value associated with an indicator is supposed to be below 5 [97,110]. Considering this, all of the values are below the upper threshold of 5, thus justifying the collinearity of indicators included in the current study (See Table 9).

**Table 9:** VIF values.

Items	VIF
**OC1**	2.478
**OC10**	1.607
**OC13**	1.340
**OC2**	2.837
**OC3**	2.663
**OC4**	2.055
**OC5**	2.035
**OC6**	2.027
**OC7**	1.624
**OC8**	1.784
**OC9**	1.995
**RA2**	1.632
**RA3**	1.522
**RA4**	1.507
**RC2**	1.598
**RC3**	1.636
**RC4**	2.083
**RC5**	2.288
**RC6**	1.836
**RC7**	1.865
**RC8**	1.430
**RC8**	1.629
**TI1**	2.713
**TI2**	3.780
**TI3**	2.444
**WFC1**	2.728
**WFC2**	2.687
**WFC3**	2.705
**WFC4**	2.570
**WFC5**	2.606
**WFC6**	2.857
**WFC7**	2.563
**WFC8**	2.526
**WOL1**	1.850
**WOL10**	1.711
**WOL12**	1.716
**WOL13**	2.178
**WOL14**	2.126
**WOL2**	1.844
**WOL3**	1.691
**WOL4**	1.550
**WOL5**	1.775
**WOL9**	1.661
**SE1**	1.291
**SE10**	1.260
**SE2**	1.842
**SE3**	1.907
**SE4**	1.883
**SE5**	1.704
**SE6**	1.539

OC: Organizational Cynicism, RA: Role Ambiguity, RC: Role Conflict, RS: Role Stressors, TI: Turnover Intentions, WFC: Work-Family Conflict, WO: Work Overload, SE: Self-Efficacy.

### Evaluation of structural model

The relationships and the respective strengths between the variables of the measurement model are defined in terms of the path coefficients and their respective magnitude. Further, the relevance of the relationships existent in the structural model is justified in terms of the significance associated with these relationships [111,112]. The values of path coefficients vary from +1 to -1. The path coefficient values closer to +1 are representative of a positive relationship between the given variables. On the contrary, the path coefficient values closer to -1, represent a negative relationship between the variables. The significance of path coefficients is assessed through the bootstrapping approach. The significance is justified in terms of the t-statistics (path coefficients divided by standard error) and the ρ-values (probability measure of erroneously discarding the null hypothesis). The critical t-values associated with the level of significance of 1%, 5%, and 10% are 2.57, 1.96, and 1.65 respectively for the two-tailed assumption [113] (See Table 10).

**Table 10:** Path coefficients.

Variables	Organizational Cynicism	Role Ambiguity	Role Conflict	Role Stressors	Turnover Intentions	Work-Family Conflict	Work Overload	Self-Efficacy
**Organizational Cynicism**					0.321			
**Role Ambiguity**				0.027				
**Role Conflict**				0.293				
**Role Stressors**	0.394				0.295			
**Turnover Intentions**								
**Work-Family Conflict**				0.435				
**Work Overload**				0.385				
**Self-Efficacy**					0.133			

**Fig 2:**
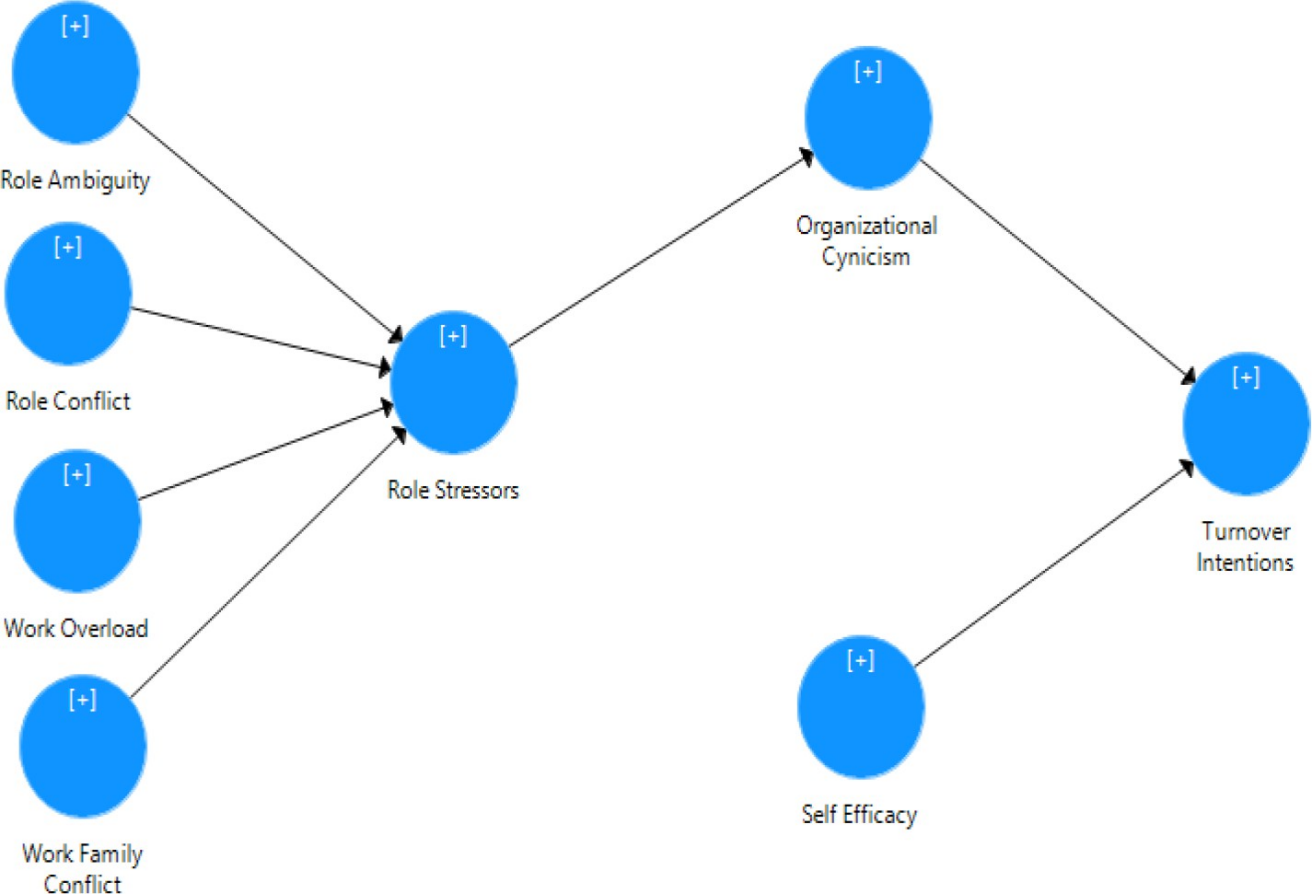
Path coefficients (SmartPLS based SEM model).

The results suggest that all the path coefficients are significant and aligned with the hypothesized model. Further, the possible variance of the variables is determined in terms of the values associated with the path coefficients. This suggests (Work-Family Conflicts-Role Stressors) as a path with the highest path coefficient value of 0.435 (See Fig 2).

### Coefficient of determination (R^2^)

The predictive accuracy of the structural model is determined in terms of R^2^. It is representative of the correlation between the actual and predictive values of a given endogenous construct. R^2^ enables explaining the possible amount of variance that can be observed in a given endogenous construct, explained by all the exogenous constructs it is linked to. The possible value of R^2^ varies between 0 to 1. A higher amount of variance and respective predictive accuracy is observed if the value is closer to 1 [114] (See Table 11).

**Table 11:** R^2^.

Variable	R Square	R Square Adjusted
**Turnover Intentions**	0.299	0.296

The R^2^ for the dependent variable of turnover intentions in the current study got evaluated being equivalent to 0.299. Which justifies the fact that the independent variables of role stressors (Role Overload, Work-family Conflicts, Role Conflicts, Role Ambiguity) and organizational cynicism explain 29.9% of the variance in the turnover intents of an individual.

### Effect size (*f*^2^)

The criterion of *f*^2^ is used to assess the size of effect an exogenous construct can put over an endogenous construct of a given model. The values of 0.02, 0.15, and 0.35 for the criterion of *f*^2^ are representative of the small, medium, and large effects depicted by the respective exogenous constructs [115,116] (See Table 12).

**Table 12:** Effect size (f^2^).

Variables	Organizational Cynicism	Role Ambiguity	Role Conflict	Role Stressors	Turnover Intentions	Work-Family Conflict	Work Overload	Self-Efficacy
**Organizational Cynicism**					0.119			
**Role Ambiguity**				0.686				
**Role Conflict**				0.234				
**Role Stressors**	0.184				0.412			
**Turnover Intentions**								
**Work-Family Conflict**				0.351				
**Work Overload**				0.455				
**Self-Efficacy**					0.022			

As per the given evaluations, all the dimensions of role stressors were found to have a large effect on the primary construct, except the work-family conflicts, which were found to have a medium effect size. Followed by which, the effect size of the independent variable of role stressors upon the mediating variable of organizational cynicism was found to be medium. While the mediatory variable itself as well had a medium effect on the dependent variable of turnover intentions. Moreover, the role stressors were found to have a large impact on turnover intentions. Lastly, self-efficacy as a moderating variable as well was found to have a medium impact on the turnover intentions of the nurses.

### Hypothesis testing

The current study proposed a total of five hypothesizes to conclude the research objectives. Out of these five hypothesizes three were of direct effect nature. The direct effects included, the impact of role stressors on the turnover intentions of an employee, the impact of role stressors upon the organizational cynicism observed in an employee, and the impact of organizational cynicism on the turnover intentions observed in an employee. Followed by which, the indirect mediatory effect of organizational cynicism was observed between the role stressors and the turnover intentions of an employee. Lastly, self-efficacy was taken into consideration as a moderator between the cynic attitudes of the nurses and their relative impact on the intent to quit.

To evaluate the direct impact of the proposed relationships along with the significance was evaluated in terms of the path coefficients and their respective significance levels. The SmartPLS algorithm along with bootstrapping with recursive iterations was used to accomplish the evaluation (See Table 13).

**Table 13:** Hypothesis testing.

Hypothesis	Path Coefficients	Significance
**H1: Role Stressors positively affect the turnover intentions of an employee.**	0.295	0.000
**H2: Role Stressors will positively affect the organizational cynicism of an employee.**	0.394	0.000
**H3: Organizational cynicism positively affect the turnover intentions of an employee.**	0.321	0.000

All the proposed relationships between the suggested variables were found significant, thus approving the proposed relationships.

### Mediation analysis

A mediatory variable is the one that causes the mediation effect between an independent variable and a dependent variable. In simpler terms, a mediator being an explanatory variable enhances the reasoning associated with the relationship between the prior mentioned variable types [117,118].

In the current study, the influences of role stressors were observed on the turnover intentions of an employee while considering organizational cynicism as a mediator.

The mediation analysis accomplished in SMART PLS is based upon the principles of Preacher and Hayes [119] as well as the bootstrapping approach that exhibits a higher level of statistical power. Prior research has indicated that while utilizing an indirect approach to test for mediation, the significance of effects depicted by exogenous variables upon the dependent variable may get lessened to some extent when a mediator is introduced in a model. While Zhao, Lynch Jr [120] argued that, hierarchical direct effect testing is not necessary to justify a mediation effect. Rather an indirect path analysis is comparatively a commended approach to justify a mediation effect. The latter approach was implemented for the current study.

Considering the proposed approach of SMART PLS to test for mediation, it was expected of exogenous variables to have a significant impact on the mediatory variable, and a mediatory variable to have a significant impact on the endogenous variable (See Table 14).

**Table 14:** Indirect effects.

	Original Sample (O)	Sample Mean (M)	Standard Deviation (STDEV)	T Statistics (|O/STDEV|)	P Values
**Role Stressors > Organizational Cynicism > Turnover Intentions**	0.117	0.121	0.024	4.791	0.000

The mediatory effect of organizational cynicism was found significant between the role of stressors and turnover intentions. Thus, the hypothesized mediatory relationship was approved for the current study.

### Moderation analysis

A moderating variable is a variable that causes an increase or decrease in the magnitude of the effect of an independent variable upon a designated dependent variable. [117,118].

In the current study, the influence of organizational cynicism was observed on the turnover intentions of an employee while considering self-efficacy as a moderator.

**Fig 3:**
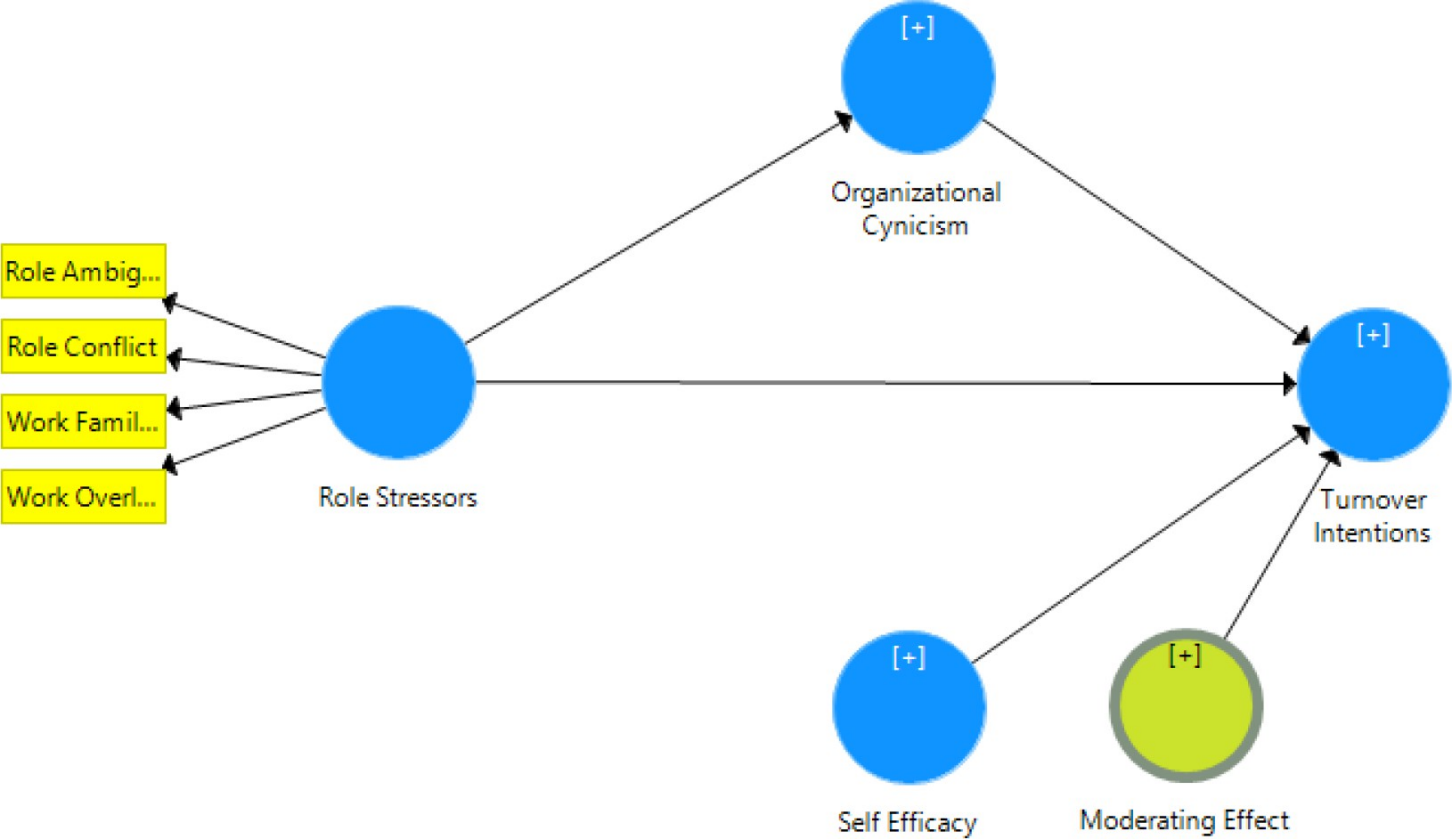
Moderation analysis (SmartPLS based SEM model).

The moderation analysis accomplished in SMART PLS is based upon the principles of Ringle, Wende [121] as well as the bootstrapping approach that exhibits a higher level of statistical power and further justifies the significance of the proposed relationship. Prior research has commended the utilization of a product approach for testing moderation. Considering which, the current study considered self-efficacy as a moderator as well as the relative product term and evaluated the respective impact on the dependent variable of followed the approach evaluates for the magnitude of the significance of effects depicted by exogenous variables upon the dependent variable of turnover intentions [121] (See Fig 3).

Considering the proposed approach of SMART PLS to test for moderation, the results depicted a significant role of self-efficacy as a moderator between the organizational cynicism attributed to the nurses and their respective intents to leave the organization. Thus, the hypothesized moderating relationship was approved for the current study (See Table 15).

**Table 15:** Moderation effect.

	Path Coefficient	P Values
**Organizational Cynicism > Self-Efficacy> Turnover Intentions**	-0.223	0.034

All the proposed hypothesizes were accepted for the current study (See Table 16).

**Table 16:** Results summary.

Index	Hypothesis	Result
**H1**	Role stressors will positively affect turnover intentions.	Accepted
**H2**	Role stressors will positively affect organizational cynicism.	Accepted
**H3**	Organizational cynicism will positively affect turnover intentions.	Accepted
**H4**	Organizational cynicism will significantly mediate the relationship between role stressors over turnover intentions.	Accepted
**H5**	Self-efficacy will significantly moderate the relationship between organizational cynicism and turnover intention.	Accepted

Conclusively all the direct effects i.e., H1, H2 & H3 were found to be significantly impacting. Furthermore, the mediating role of organizational cynicism between role stressors and turnover intentions was proved to be significant as well. Lastly, the moderating effect of self-efficacy to control the magnitude of relational strength between organizational cynicism and turnover intentions too was found significantly accepted.

## Discussion & conclusion

The current study while considering the priorly identified research gaps has accomplished to conceptually explored the cumulative effects of four primary dimensions of role stressors in instigating the intents of turnover in an employed individual. The said effect is further evaluated in terms of the development of cynical attitudes within an employee towards his/her coworkers and the organization. Further, the current study evaluated the compensating effect of self-efficacy while considering its role as a moderator between organizational cynicism and the turnover intentions associated with the nurses. In terms of accomplishing the contextual gap, the current study took into account the healthcare industry of Pakistan. That over the years has remained unattended in terms of the development and implementation of behavioral reforms.

As per the deductions of the current study, it was observed that role stressors i.e. (role ambiguity, role conflict, work-family conflict, and role overload) had an inclined influence on the intent of the nurses to quit their jobs. Similar phenomena have been observed in the study conducted by Evans and Steptoe [122] upon 588 nurses and 387 accountants, which showed that stress inflicted upon an individual either from professional routine or personal life is probable to develop psychological problems for an individual, which may lead towards long-term physical ailments. This may further draw the professionals away from their employing organization. Tziner, Rabenu [123] in their study conducted on 124 healthcare physicians as well indicated stress to be a very probable cause to cause discomfort in the professionals and weaken their commitment of theirs to their organization. Thus, causing the development of intents to turnover. Likewise in one of the most recent studies conducted by Harun, Mahmood [124] on Malaysian doctors working in the public sector indicated the role stressors as an instigating factor leading to turnover of an individual. Therefore, the healthcare management is encouraged to consistently monitor if any of the individuals have been under stress for long periods either because of professional commitments or personal ones. Moreover, the employed individuals must be granted access to counseling programs and given incentives in compensation for their efforts. Doing so will enable the healthcare workers to be able to get the necessary help in time and have their goodwill remain maintained towards the employing organization [125,126].

Further, the current study reported the role stressors faced by the nurses cause the employees to become more cynic towards their organization. Not only that but also the cynic portrayal of attitude was observed towards other colleagues and service consumers. This outcome is supported by the systematic review conducted by Lizano [127], who took into account 19 relevant studies spanned across the period of 1970 to 2014 to evaluate the strain experienced by the service industry professionals and its further translation in terms of cynic attitudes towards coworkers or family. The results of his study suggested a common pattern in terms of the strain affecting the professionals adversely and disrupting their work-life balance. Akin to which, Tong, Oh [128] in their research conducted on 163 individuals from various professions suggested the role of stressors to develop cynical behaviors. Moreover, the current study recognized the fact that the nurses facing role stressors and subsequently becoming cynical, were also observed to develop the intent to leave the organization. These intents to leave the organization to a better extent were countered by the self-efficacious behavior of the nurses, which not only helped them to cope with the self-developed cynic behaviors but also enabled them to remain committed to their organization [129,130]. Akin to this, a study conducted by Chong and Monroe [131] on 368 junior accountants serving in the public sector suggested that strain experienced by the employed individuals results in the development of adverse attitudes of employees towards their organization which further leads to the initiation of turnover intentions. Though Park and Jung [132] in their study conducted on 555 individuals from various professional backgrounds indicated that if employees get to be equipped with self-efficacious attitudes they develop resilience towards intending to leave their employing organization and are less prone to get effected by the adverse circumstances faced in professional and personal capacity. Considering which, it has been commended for the health care institutions to provide their employees with manageable workloads, as well as carter their needs in terms of supporting their personal requirements; as much as possible. Moreover, the managers of healthcare institutions are encouraged to initiate on-job training programs for the employees. This will enable healthcare workers to remain equipped with the up-to-date knowledge, in order to tackle the ever-growing job demands and therefore be able to fulfill the job expectations. Also, it will give an effective chance to the managers to learn about the employed individuals and develop management strategies, while keeping considering those learnings [133–135].

In terms of the limitations and respective future directions associated with the current study few considerations can be taken into account. The current study took the role stressors as the primary factors leading to the turnover intentions of healthcare workers. Also, there can other factors that may lead to adverse organizational outcomes such as turnover intentions, and therefore can further be explored. Referred to this, considerations including leadership challenges, lack of motivation, and unhealthy working environment, professional incompetence, and more can be taken into account. Moreover, in the contextual aspect, a similar research model as proposed in the current study can be tested in other relevant industries within the country facing similar critical levels of stress. Also, the replication of the currently proposed research model can be reevaluated in the context of the healthcare industry of other developing nations. Both of these will result in drawing a comparative comparison in determining the stress experienced by individuals employed in other sectors or regions. Thus, leading to lining out common practices that can be adopted to mitigate the adverse outcomes of such factors.

## Supporting information

S1 Survey Contains all the research items used in the current study. (DOCX)
