# Supplementary material for: Impact role stress on turnover intentions of Pakistan’s healthcare workers: Mediating and moderating role of organizational cynicism and self-efficacy
Source: PLoS One. 2022 Dec 15;17(12):e0279075. doi: 10.1371/journal.pone.0279075 (PMC9754294; doi:10.1371/journal.pone.0279075)
Supplement: S1 Survey — (DOCX) [file pone.0279075.s001.docx]

**Survey Form**

**Part A: Demographics**

| **Age** | | [ 1] 20-30 | [ 2] 30-40 |
| --- | --- | --- | --- |
|  | | [ 3] 40-50 | [ 4] 50 and above |
| **Gender** | [1] Male | | [2] Female |
| **Tenure** | | [ 1] Less than 1 year | [ 2] 1 – 2 years |
|  | | [ 3] 2 – 5 years | [ 4] 5 – 10 years |
|  | | [ 5] 10 & above |  |
| **Sector** | | [ 1] Public | [ 2] Private |
|  | | | |
| **Employment Status** | | [ 1] Permanent | [ 2] Contractual |

|  |  | Strongly Disagree | Disagree | Neutral | Agree | Strongly Agree |
| --- | --- | --- | --- | --- | --- | --- |
| **Part B: Organizational Cynicism** | | | | | | |
| 1 | When I think about my organization, I experience irritation. | -1- | -2- | -3- | -4- | -5- |
| 2 | When I think about my organization, I experience aggravation. | -1- | -2- | -3- | -4- | -5- |
| 3 | When I think about my organization, I experience tension. | -1- | -2- | -3- | -4- | -5- |
| 4 | When I think about my organization, I experience anxiety. | -1- | -2- | -3- | -4- | -5- |
| 5 | I believe that my organization says one thing and does another. | -1- | -2- | -3- | -4- | -5- |
| 6 | My organization's policies, goals, and practices do not give clear picture to its employees. | -1- | -2- | -3- | -4- | -5- |
| 7 | When my organization says it's going to do something, I wonder if it will really happen. | -1- | -2- | -3- | -4- | -5- |
| 8 | My organization expects one thing of its employees, but rewards another. | -1- | -2- | -3- | -4- | -5- |
| 9 | I find little similarity between what my organization say it will do and what it actually does. | -1- | -2- | -3- | -4- | -5- |
| 10 | I criticize about how things happen at my organization and also tell friends outside the organization. | -1- | -2- | -3- | -4- | -5- |
| 11 | I understand the feelings of my colleagues just by looking at their faces. | -1- | -2- | -3- | -4- | -5- |
| 12 | I talk to others about the rules and regulations of my organization that mostly annoy me. | -1- | -2- | -3- | -4- | -5- |
| 13 | I criticize my organization's practices and policies with others. | -1- | -2- | -3- | -4- | -5- |
| **Part C: Turnover Intention** | | | | | | |
| 14 | As soon as I can find a better job, I’ll leave the organization. | -1- | -2- | -3- | -4- | -5- |
| 15 | I am actively looking for a job outside the organization. | -1- | -2- | -3- | -4- | -5- |
| 16 | I am seriously thinking of quitting my job. | -1- | -2- | -3- | -4- | -5- |
| **Part D: Role Stressors** | | | | | | |
| *Role Overload* | |  |  |  |  |  |
| 17 | I have things to do which I don’t really have the time and energy for. | -1- | -2- | -3- | -4- | -5- |
| 18 | There are too many demands on my time. | -1- | -2- | -3- | -4- | -5- |
| 19 | I need more hours in the day to do all the things which are expected of me. | -1- | -2- | -3- | -4- | -5- |
| 20 | I never caught up in order to neglect my duty. | -1- | -2- | -3- | -4- | -5- |
| 21 | I don’t ever seem to have any time for myself. | -1- | -2- | -3- | -4- | -5- |
| 22 | I do not fulfill the expectations of all regarding my work. | -1- | -2- | -3- | -4- | -5- |
| 23 | Sometimes I feel as if there are not enough hours in the day. | -1- | -2- | -3- | -4- | -5- |
| 24 | Many times, I have to cancel commitments. | -1- | -2- | -3- | -4- | -5- |
| 25 | I take tension in order to be able to finish everything I have to do. | -1- | -2- | -3- | -4- | -5- |
| 26 | I am more committed towards my work than some other colleagues I know. | -1- | -2- | -3- | -4- | -5- |
| 27 | I find myself having to prepare priority lists (lists which tell me which things I should do first) to get done all the things I have to do. Otherwise, I forget. | -1- | -2- | -3- | -4- | -5- |
| 28 | I feel I have to do things hastily and maybe less carefully in order to get everything done. | -1- | -2- | -3- | -4- | -5- |
| 29 | I just can’t find the energy in me to do all the things expected of me. | -1- | -2- | -3- | -4- | -5- |
| 30 | I have things to do which I don’t really have the time and energy for. | -1- | -2- | -3- | -4- | -5- |
| *Work Family Conflict* | |  |  |  |  |  |
| 31 | As I have many tasks and responsibilities at work, I cannot spend time with my family. | -1- | -2- | -3- | -4- | -5- |
| 32 | Physical and mental fatigue at work makes my responsibilities at home difficult. | -1- | -2- | -3- | -4- | -5- |
| 33 | Fatigue at work decreases my tolerance towards family members. | -1- | -2- | -3- | -4- | -5- |
| 34 | My duties at the workplace make me feel tired while performing my responsibilities at home. | -1- | -2- | -3- | -4- | -5- |
| 35 | I cannot spare time for house works due to my job. | -1- | -2- | -3- | -4- | -5- |
| 36 | My work life reduces my efforts necessary for my responsibilities at home. | -1- | -2- | -3- | -4- | -5- |
| 37 | I continue thinking about a problem I faced in the workplace. | -1- | -2- | -3- | -4- | -5- |
| 38 | A problem at the work life makes me stressed and uneasy at home. | -1- | -2- | -3- | -4- | -5- |
| 39 | My duties and responsibilities at work get ahead of my family life. | -1- | -2- | -3- | -4- | -5- |
| *Role Conflict* | |  |  |  |  |  |
| 40 | I have to do things that should be done differently. | -1- | -2- | -3- | -4- | -5- |
| 41 | I receive an assignment without the manpower to complete it. | -1- | -2- | -3- | -4- | -5- |
| 42 | I have to violate a rule or policy in order to carry out an assignment. | -1- | -2- | -3- | -4- | -5- |
| 43 | I have to work with two or more groups who operate quite differently. | -1- | -2- | -3- | -4- | -5- |
| 44 | I receive incompatible requests from two or more people. | -1- | -2- | -3- | -4- | -5- |
| 45 | I do things that are apt to be accepted by one person and not accepted by others. | -1- | -2- | -3- | -4- | -5- |
| 46 | I receive an assignment without adequate resources and material to execute it. | -1- | -2- | -3- | -4- | -5- |
| 47 | I work on unnecessary things. | -1- | -2- | -3- | -4- | -5- |
| *Role Ambiguity* | |  |  |  |  |  |
| 48 | I am confident about how much authority I have. | -1- | -2- | -3- | -4- | -5- |
| 49 | I have clear, planned objectives for my job. | -1- | -2- | -3- | -4- | -5- |
| 50 | I know that I have divided my time properly. | -1- | -2- | -3- | -4- | -5- |
| 51 | I know what my responsibilities are. | -1- | -2- | -3- | -4- | -5- |
| 52 | I know exactly what is expected of me. | -1- | -2- | -3- | -4- | -5- |
| 53 | I receive clear explanations of what has to be done. | -1- | -2- | -3- | -4- | -5- |
| **Part E: Organizational Cynicism** | | | | | | |
| 54 | I can always manage to solve difficult problems if I try hard enough. | -1- | -2- | -3- | -4- | -5- |
| 55 | If someone opposes me, I can find the means and ways to get what I want. | -1- | -2- | -3- | -4- | -5- |
| 56 | It is easy for me to stick to my aims and accomplish my goals. | -1- | -2- | -3- | -4- | -5- |
| 57 | I am confident that I could deal efficiently with unexpected events. | -1- | -2- | -3- | -4- | -5- |
| 58 | Thanks to my resourcefulness, I know how to handle unforeseen situations. | -1- | -2- | -3- | -4- | -5- |
| 59 | I can solve most problems if I invest the necessary effort. | -1- | -2- | -3- | -4- | -5- |
| 60 | I can remain calm when facing difficulties because I can rely on my coping abilities. | -1- | -2- | -3- | -4- | -5- |
